# A Prediction Model for Various Treatment Pathways of Upper Extremity in Tetraplegia

**DOI:** 10.3389/fresc.2022.889577

**Published:** 2022-06-30

**Authors:** Ines Bersch, Jörg Krebs, Jan Fridén

**Affiliations:** Swiss Paraplegic Centre, Nottwil, Switzerland

**Keywords:** motor points, outcome prediction, electrical stimulation, upper extremities, tetraplegia

## Abstract

Upper extremity function is essential for the autonomy in patients with cervical spinal cord injuries and consequently a focus of the rehabilitation and treatment efforts. Routinely, an individualized treatment plan is proposed to the patient by an interprofessional team. It dichotomizes into a conservative and a surgical treatment pathway. To select an optimal pathway, it is important to define predictors that substantiate the treatment strategy. Apart from standard assessments (Standards for Neurological Classification of Spinal Cord Injury (ISNCSCI), the manual muscle test (MRC), and lower motoneuron integrity of key actuators for hand function performed by motor point (MP) mapping might serve as a possible predictor. Type of damage (upper motor neuron (UMN) or lower motor neuron (LMN) lesion) influences hand posture and thus treatment strategy as positioning and splinting of fingers, hands, arms, and surgical reconstructive procedures (muscle-tendon or nerve transfers) in choice and timing of intervention. For this purpose, an analysis of a database comprising 220 patients with cervical spinal cord injury is used. It includes ISNCSCI, MRC, and MP mapping of defined muscles at selected time points after injury. The ordinal regression analysis performed indicates that MP and ASIA impairment scale (AIS) act as predictors of muscle strength acquisition. In accordance with the innervation status defined by MP, electrical stimulation (ES) is executed either *via* nerve or direct muscle stimulation as a supplementary therapy to the traditional occupational and physiotherapeutic treatment methods. Depending on the objective, ES is applied for motor learning, strengthening, or maintenance of muscle contractile properties. By employing ES, hand and arm function can be predicted by MP and AIS and used as the basis for providing an individualized treatment plan.

## Introduction

Restoration of hand and arm function is one of the therapeutic priorities in the rehabilitation of patients with cervical spinal cord injuries (cSCIs). The hand treatment commences immediately after injury in the intensive care unit. It consists of splinting and positioning, passive mobilization techniques, functional training, and ES (1). The interventions aim for the prevention of joint contractures, enhancement of reinnervation, strengthening of voluntary active musculature, and development of the hand shape that allows the greatest functionality for any specific level of the lesion.

In addition to the traditional therapeutic treatment of the tetraplegic hand, there is also a choice of surgical procedures such as muscle tendon and nerve transfers that can improve hand–arm function. Nerve transfers in particular are time-sensitive if the recipient's muscle lacks intact lower motor neurons (2, 3). These muscles should be stimulated as early as possible by direct electrical muscle stimulation with long pulse widths (4).

The interprofessional team decides which procedure should be considered and provides the greatest possible benefit to the individual patient. The rationale for the decision is based on the results of a series of clinical assessments and examinations, which ultimately form a decision pathway for the planned treatment. Thus, standardized examinations to define the level of lesion, manual testing of muscle strength, and examination of the integrity of the lower motor neuron in key muscles of the upper limb are part of the routinely performed diagnostic tests (5). The level of lesion is determined by the ASIA impairment score (AIS).

Testing of the motor points in key muscles responsible for grasping and moving in space of the upper extremity by the means of ES has become established in recent years (6, 7). Employing short pulse duration electrostimulation as a diagnostic tool allows for the identification of LMN integrity in defined muscles. Mapping of the dorsal and palmar side of the forearm includes standardized reproducible stimulation points that excite the muscle with the lowest intensity and the greatest selectivity using surface electrical stimulation. Voluntary motor activity tested by the manual muscle status provides an initial indication of the expected functional outcome of hand–arm function. The manual muscle testing of the upper limb is based on the British Medical Research Council Scale and assessed at monthly intervals (8). The test is based on an ordinal scale, 0–5, and serves as a trajectory parameter in the neurological recovery of voluntary motor function. Hence, ISNCSCI, MRC, and MP mapping, in addition to the clinical examination, establish the treatment strategy. Neurophysiological diagnostics can be consulted and, if a surgical procedure is considered, the International Classification for Surgery of the Hand in Tetraplegia applies (9).

The information obtained from all the tests is used to define treatment priorities. ES is used in therapeutic treatment as an additional therapy that may be combined with traditional conservative ergo- and physiotherapy. Since ES with long and short pulses has been recognized as a treatment method in rehabilitation, there is scientific evidence of its effectiveness (10). Afferent and efferent stimulation for neuromodulation (11, 12), motor learning, and/or relearning of functions (13–15), strengthening of individual muscles or muscle groups (16, 17), contracture prophylaxis and maintenance or improvement of muscle properties (18–20) are essential for the treatment of the upper limb in tetraplegia.

Motor learning includes mainly the acquisition or reacquisition of motor skills. If these cannot be learned due to manifest neurological deficits, adaptations occur throughout the learning process of movements (21). During neurological recovery after cSCI, the maintenance of the cortical representation of hand function can support the relearning process without compensatory movement strategies. Several studies in neurorehabilitation show that the combination of ES and task-oriented training is superior to task-oriented training alone (13, 22–24). However, either EMG-triggered stimulation (25) or cyclical stimulation (26), or somatosensory stimulation (27, 28) in combination with the functional task-oriented stimulation can be administered. Furthermore, during the regeneration of functions, the influence of brain-derived neurotropic factor (BDNF) needs to be considered. Combining traditional therapeutic treatment approaches with ES can increase the release of BDNF (29). In animal studies, it has been shown that ES-elicited muscle contraction in response to the associated movement and increased BDNF levels in the spinal cord and in the muscle (30). These results are indicators that ES in motor learning can promote neurological recovery and is recommended to be applied in principle.

In addition, strengthening of muscles or muscle groups can be successfully performed and supported by ES. Several studies in individuals with cSCI have shown that it is possible to increase torque and power output by FES-supported exercises in upper extremities if the LMN is intact (17, 31, 32). In addition, AIS, the type of the lesion according to the muscles to be stimulated (innervated, partially innervated/denervated, and denervated) and the time after injury should be considered. The latter affects the muscle fiber shift (18), as evidenced by the increase in the proportion of fast glycolytic and fatigable type IIB fibers and a decrease in slow oxidative and fatigue-resistant type I fibers. In clinical practice, it should therefore be kept in mind that muscle fiber shift from type I to type II as well as muscle atrophy influences endurance and fatigability of muscle (33). Atrophied muscles in chronic SCI wear out earlier and require lower frequency stimulation at the beginning of the ES session (33, 34).

Following an LMN lesion, the maintenance and reversal of muscle properties are crucial. Denervation atrophy results in a decrease in muscle fiber diameter and a partial to complete transformation of muscle tissue into connective and adipose tissue (35). In a previous study, we demonstrated that in early as well as chronic damage to the LMN, electrostimulation with long pulses enabled maintenance and restoration of muscle properties (20). In accordance with our results and with the research carried out on the muscles of the lower limb (36), an early start of this type of stimulation is recommended. ES with long pulses on denervated upper limb muscles preserves contractile muscle tissue. It provides a decisive base for possible reinnervation and surgical interventions such as nerve transfers where the morphology of the recipient's muscle is crucial for a successful outcome of the procedure (4).

The aim of this report is to evaluate possible predictors, of muscle strength based on standardized measurement as ISNCSCI, MRC, and MP mapping. We hypothesize that based on the identified predictors, conservative and surgical procedures and also as protocols for ES can be defined individually within the first 2 months after injury.

## Methods

To identify possible predictors relevant for the decision of the individualized treatment pathway, a data collection that currently comprises 220 patients with cSCI was analyzed. A data set was defined as complete if the MP was performed 4–8 weeks post injury and MRC values have not been >3 at the time of testing and were available at 24 weeks post injury.

All the data collected for flexor digitorum profundus (FDP) and extensor digitorum communis (EDC) muscles and also those of the brachioradialis (BR) were extracted. Since MCR is not performed for BR, only MP data were used in that case. To identify possible early predictors for the shape and functionality of the hand, finger flexors (FDPs) and extensors (EDC) were additionally analyzed with AIS, MP, NLI, and MRC for possible interdependencies. Collectively, these diagnostic elements form a decision pathway, for example, whether an early nerve transfer should be considered.

### The Rationale for the Selection of Muscles

The existing database includes testing of selected intrinsic and extrinsic hand muscles relevant to hand function, and also muscles of the upper arm. The present study focused on three muscles, the EDC and FDP as well as the BR.

The concomitantly acting EDC and FDP muscles are important actuators in the development of the function and shape of a tetraplegic hand. They are responsible for finger extension and finger flexion. The muscular imbalance between them results in deformities such as claw hands or closed fists. In addition, their interaction is decisive for the development of the tenodesis grip.

The excitability of the motoneurons to BR is used to decide whether it can be used as a salvage donor for reanimation of, for example, finger flexors or thumb flexors (37). The selective strengthening of BR, however, presents a technical challenge. Sufficient strength is needed for ensuing muscle-tendon transfer (38).

### Stimulation Equipment for Diagnostics and Treatment

A two-channel nerve stimulator with continuously adjustable amplitude is recommended for the use of ES as a diagnostic tool. The parameters used for testing are in the range of 250–300 μs pulse width and at 35 Hz. A sufficient amplitude to elicit a contraction in the muscles of the upper extremity is between 20 and 80 mA (4).

Commercially available nerve stimulators are used for the treatment. At best, they offer the possibility of individual programming, i.e., pulse duration, frequency, amplitude, and duty-cycle of stimulation can be programmed individually.

A special stimulator is recommended for direct muscle stimulation with long pulses. It permits sufficiently high-stimulation intensity and tetanic contractions. The recommended parameter composition to influence muscle morphology comprises: 1. biphasic rectangular shape and 20 to 40 ms pulse duration with bursts of 2 s (2s pause), 2. 20 Hz and amplitudes 30–80 mA for the upper limb muscles (39, 40).

### Application of ES Diagnostics

Classification of motor neuron lesions in upper limb muscles is required for further effective treatment with ES. The MP mapping provides a feasible and reliable method for determining the integrity of the LMN. In accordance with the MRC, the MP mapping classifies a muscle as denervated if no contraction can be achieved under stimulation, as partially innervated/denervated if no full range of joint motion can be elicited by stimulation, and as innervated if a full range of motion can be provoked (6).

Patients with cSCI are likely to have lower motor neuron damages at the level of injury and one to two segments above and/or below. Muscles, whose segmental innervation originates within these segments may be affected (7, 41). Once the innervation status has been characterized, a decision can be made whether stimulation should be performed directly *via* muscle with long (ms) or *via* nerve with short impulses (μs) or in a combination of alternating long and short impulses. The latter applies to muscles with partial damage to the LMN.

Furthermore, the objective of treatment determines the type of stimulation and whether and how stimulation can be integrated into the traditional therapeutic treatment, and also the number of stimulation sessions per day and week.

In the neurotization procedures, the recipient muscle's LMN conduction properties guide the planning (42) while for muscle-tendon transfers, the functional integrity of the donor is the determining factor. In case of partial LMN damage, nerve transfer may be deterred due to the structural transformation of contractile components to connective and adipose tissue.

### Statistical Methods

An ordinal regression analysis of the odds ratios (ORs) was performed to evaluate the effect of possible predictors on MRC (dependent variable). The following predictors were included: MP, AIS, and NLI. All the variables were entered in a single step. For categorical predictors, the odds ratios with 95% CIs were calculated in comparison to a reference category. The reference category refers to the one category of a certain predictor to which the other categories of the same predictor are compared to. Ordinal regression analysis was performed for FDP and EDC muscles. Statistical analyses were performed using SPSS software (Version 25, IBM, Somers, NY, USA). The level of significance was set at *p* ≤ 0.05.

Descriptive analyses were used to illustrate the integrity of LMN of BR relative to segmental innervation and level of the lesion.

## Results

Complete data sets including MP testing 4–8 weeks post injury, a temporally correlated ISNCSCI classification, MRC at 24 weeks post injury of 86 patients, 75 men/11 women, with a mean age of 46.5 ± 20 years, respectively, 172 FDP and EDC muscles were analyzed. The levels of lesion ranged from C3 to C8 AIS A–D (Table 1).

**Table 1:** Distribution of the neurological level of injury and ASIA impairment scale for the patient cohort studied.

**AIS**	**A**	**B**	**C**	**D**
**NLI**				
C3	3	1	1	5
C4	6	3	5	11
C5	9	6	4	9
C6	8	2	3	4
C7	-	-	2	2
C8	1	-	-	1

*A = Complete: No motor or sensory function is preserved in the sacral segments S4–S5*.

*B = Incomplete: Sensory but not motor function is preserved below the neurological level and includes the sacral segments S4–S5*.

*C = Incomplete: Motor function is preserved below the neurological level, and more than half of key muscles below the neurological level have a muscle grade <3*.

*D = Incomplete: Motor function is preserved below the neurological level, and at least half of key muscles below the neurological level have a muscle grade of three or more*.

*NLI, neurological level of injury; AIS, ASIA impairment scale; C, cervical*.

The ordinal regression analysis identified the MP status 4–8 weeks post injury and the AIS as significant predictors of expected muscle strength at 24 weeks after injury. Hereby, MRC was defined as the dependent variable. NLI and age did not appear as significant predictors. Calculated odds ratios reflect the increased probability of a muscle strength improvement with innervated MP or AIS C–D in the FDP (Table 2) and EDC (Table 3) at 24 weeks after injury.

**Table 2:** Results of ordinal regression analysis for flexor digitorum profundus.

	**95% confidence interval**			
	**Level of significance**	**Odds ratio**	**Lower**	**Upper**
MP innervated	<0.001	6.759	2.550	18.829
MP partially	0.24	2.052	0.612	6.833
denervated				
MP denervated	Reference category			
AIS A	Reference category			
AIS B	0.005	5.028	1.642	15.882
AIS C	0.027	3.622	1.169	11.561
AIS D	<0.001	28.439	9.760	88.922
NLI C2	0.12	0.085	0.002	1.585
NLI C3	0.07	0.125	0.011	1.079
NLI C4	0.04	0.118	0.013	0.847
NLI C5	0.7	0.701	0.074	5.260
NLI C6	0.4	0.379	0.038	2.990
NLI C7	Reference category			
Age	0.2	1.013	0.994	1.033

*The MRC of the FDP was the dependent variable for the ordinal regression analysis. MP and AIS were significant predictors. Level of significance p ≤ 0.05*.

*MRC, manual muscle test based on the Medical Research Council Scale; FDP, flexor digitorum profundus; MP, motor point; AIS, ASIA impairment scale; NLI, neurological level of injury*.

**Table 3:** Results of ordinal regression analysis for the extensor digitorum communis.

	**95% confidence interval**			
		**Odds**		
	**Level of significance**	**ratio**	**Lower**	**Upper**
MP innervated	0.001	0.207	0.083	0.498
MP partially	0.068	0.324	0.093	1.071
denervated				
MP denervated	Reference category			
AIS A	Reference category			
AIS B	0.005	5.028	1.642	15.882
AIS C	0.027	3.622	1.169	11.561
AIS D	<0.001	28.439	9.760	88.922
NLI C2	0.21	0.128	0.003	2.652
NLI C3	0.22	0.233	0.021	2.191
NLI C4	0.24	0.273	0.028	2.178
NLI C5	0.99	0.996	0.107	8.132
NLI C6	0.93	0.900	0.087	8.104
NLI C7	Reference category			
Age	0.11	1.015	0.997	1.034

*The MRC of the EDC was the dependent variable for the ordinal regression analysis. MP and AIS were significant predictors. Level of significance p ≤ 0.05*.

*EDC, extensor digitorum communis; MP, motor point; AIS, ASIA impairment scale; NLI, neurological level of injury*.

For BR (54 patients, 40 men/14 women), 108 BR muscles were analyzed. In total, 48 patients had a traumatic cSCI, five patients with GBS syndrome, and one had sarcoidosis that inflicted tetraplegia. The mean age was 54 ± 18 years.

**Figure 1:**
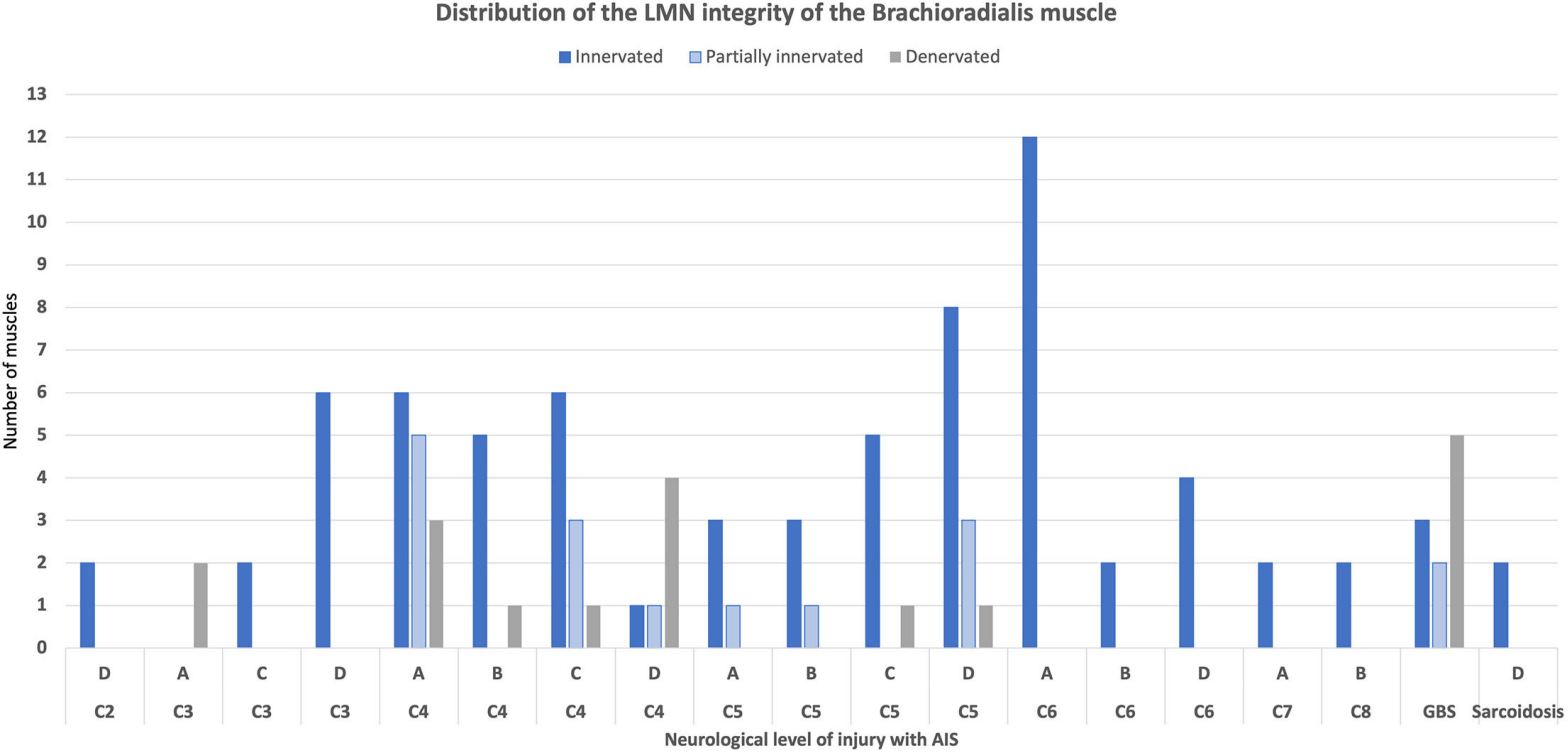
Innervation pattern of the brachioradialis muscle. GBS, Guillain–Barré Syndrome.

In total, 68.5% of the BR (74/108) showed an intact LMN and were classified as innervated whereas 14.8% (16/108) were identified as partially innervated and 16.6 % (18/108) as denervated (Figure 1).

Based on MP testing, four scenarios can arise for the two extrinsic finger muscles studied.

**Figure 2:**
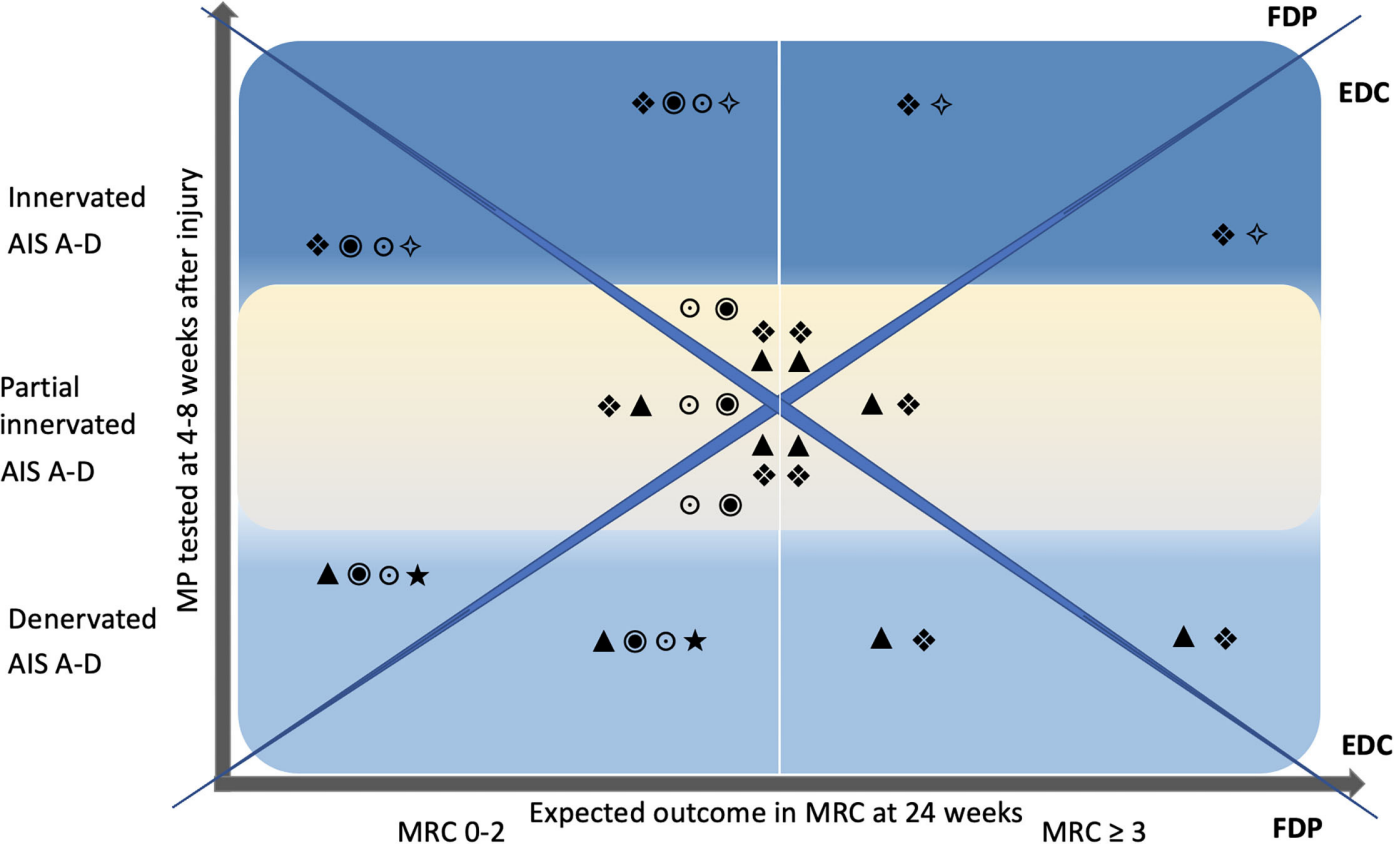
Road map. The Y-axis represents the single motor point testing 4–8 weeks after injury and is divided into the categories denervated (bright blue), partially innervated (yellow), and innervated (blue), each for AIS A–D. X-axis denotes the expected outcome of voluntary motor function tested with the MRC 24 weeks after injury in relation to the motor point innervation pattern. A vertical white line in the center of the graph divides MRC into the two categories 0–2 and ≥3. The diagonals split the graph into upper and lower as well as right and left triangles. The upper and lower triangles represent FDP and the right and left triangles to represent EDC. Inset symbols suggest individualized interventions as early as 4–8 weeks after injury. MP, motor point; MRC, manual muscle test, British Medical Research Council Scale; FDP, flexor digitorum profundus; EDC, extensor digitorum communis; •, nerve transfer, ▴, long pulse stimulation, (LMN) lesion; ❖, functional electrical stimulation with task-specific exercise (motor learning); ⋆, splinting; *, strengthening with ES; ○, passive mobilization.

Scenario 1: both muscles show signs of denervation. Scenario 2: both muscles are innervated. Scenario 3: the FDP is innervated or partially innervated and the EDC is denervated. Finally, in scenario 4: the EDC is innervated or partially innervated and the FDP is denervated. Based on the MP test results and the AIS classification, treatment strategies can be defined. A rough road map (Figure 2) can assist in the selection of treatments that should be initiated early after cSCI. The innervation pattern of MP and the AIS collected 4–8 weeks after cSCI, are predictors for the development of muscle strength at 24 weeks. This allows to include treatment procedures such as nerve transfers early in the rehabilitation process. Furthermore, electrical stimulation can be selected specifically in the classical therapeutic treatment and used for structural and functional improvements (Table 4).

**Table 4:** Presentation of four different innervation patterns and the corresponding treatment recommendation.

	**Scenario 1**	**Scenario 2**	**Scenario 3**	**Scenario 4**
MP	EDC denervated FDP denervated	EDC innervated FDP innervated	EDC denervated FDP innervated	EDC innervated FDP denervated
Clinical appearance	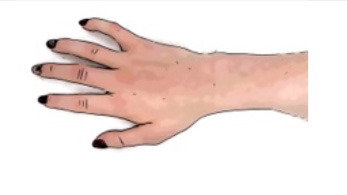	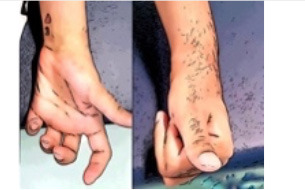	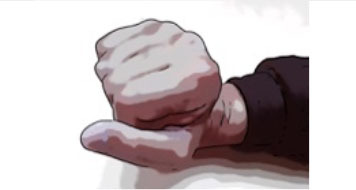	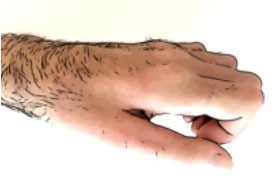
Treatment	Passive mobilization techniques to reduce risk of contracture due to denervation atrophy	Task-specific training with FES based on motor learning principles, EMG -triggered ES	Passive mobilization techniques to reduce risk of contracture mainly on the EDC followed by ES of the wrist extensors	Passive mobilization techniques to reduce risk of claw hand
	ES of denervated muscles to maintain contractile muscle tissue	Strengthening supported with ES of both EDC and FDP	Strengthening supported with ES mainly of the wrist extensors after successful mobilization of the EDC	ES of denervated flexors to maintain mobility of the MCP and PIP joints. ES of the intrinsic muscles of the hand to avoid intrinsic tightness
	Alternate positioning or splinting	Splinting not generally necessary	Splinting overnight if necessary	No splinting to avoid external stimulus applied on the muscle spindles, for example pressure or stretch on the muscle belly
	Evaluation of nerve transfer in a prompt timeframe	Information about reconstructive surgical procedures	Evaluation of reconstructive surgical procedures such as muscle-tendon or nerve transfers	Evaluation of reconstructive surgical procedures such as muscle-tendon or nerve transfers

*MP, motor point; EDC, extensor digitorum communis; FDP, flexor digitorum profundus, FES, functional electrical stimulation; ES, electrical stimulation; MCP, metacarpo-phalangeal joints; PIP, proximal interphalangeal joints*.

## Discussion

This study indicated that MP and AIS assessed 4–8 weeks after cSCI predicts the muscle strength of FDP and EDC at 24 weeks after injury. Furthermore, BR data showed a relatively high proportion of LMN lesions even when the core of damage was not at the level of segmental innervation of the muscle. Taken together, these findings are decisive for setting up an early and individualized treatment plan as well as to project the expected outcome.

The distribution of lesion levels demonstrated the highest number of lesions in the C5 and C6 categories, thus, matching the patient group traditionally targeted for therapeutic treatment to develop a tenodesis grasp. Splinting and positioning are designed to shorten the long finger flexors and the hand is then treated and trained with this goal during passive mobilization and the first functional exercises. Ultimately, the patients should achieve closure of the hand through wrist extension and opening *via* wrist flexion. Thus, a basic grasp and release would, in theory, be achieved. However, the clinical observations have shown that the level of the lesion including AIS alone does not provide enough support for this strategy to successfully reach a useful tenodesis grasp and release function. Despite consistent standardized treatment with splint fitting, positioning, and passive mobilization over 12 weeks, claw hands, contractures, and/or inadequate closing or opening of the fingers may occur. This fact necessitates the inclusion of other neurological factors and considerations for individualized treatment schedules.

The presence or absence of lower motoneuron integrity in the FDP and EDC may help to better stratify and individualize treatment (4, 41). If the LMN is intact in both the FDP and EDC 4–8 weeks post injury, development or improvement in muscle strength can be expected at 24 weeks despite lack of voluntary motor function at that time. This likelihood increases in patients with the AIS is C or D. For treatment, this implies that early FES supplement motor learning should be done in combination with grasp and release exercises and that positioning and splinting for producing tenodesis grasp can be disregarded.

**Figure 3:**
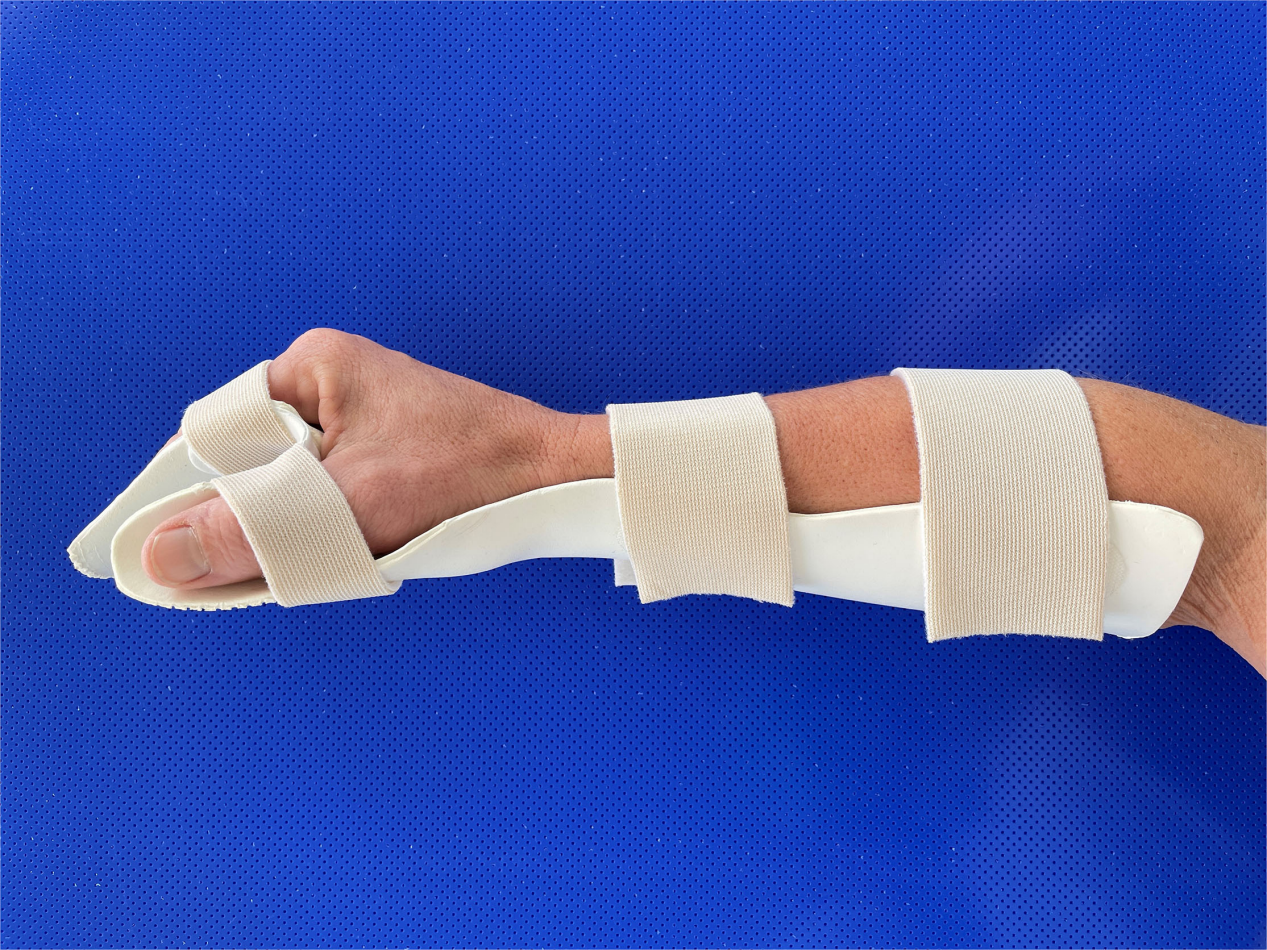
Splint to ensure the intrinsic plus position of the hand.

In contrast, if there is a lesion of the LMN in both flexor and extensor muscles, other early treatment strategies should be considered. Denervated muscles transform into connective and fatty tissues. Subsequently, the visco-elastic properties of the muscle will be lost, and muscle contractures evolve. In order to counteract such development, splinting, or positioning of the hand and wrist in the “safe” intrinsic plus position (Figure 3) for several hours daily should be reconsidered. Applying ES with the long pulses can supplement the splint treatment and maintain the contractile properties of the muscle (43).

A mixed LMN lesion pattern occurs frequently among finger flexors and extensors. If the FDPs have intact LMNs and the EDCs do not, the development of a tenodesis grasp is likely to occur with correct hand positioning. The flaccid paralysis of the finger extensor allows the finger flexors to shorten well because of their intact reflex arcs. In the selected cases, flexors may also develop voluntary motor activity supported by FES of the flexor muscles during task-specific exercises (44).

Depending upon the extent of voluntary motor function in the recipient's muscle, reconstructive nerve transfers such as SPIN (supinator donor nerve to recipient posterior interosseus nerve for finger extension) and AIN (brachial donor nerve to recipient anterior interosseus nerve for finger flexion) are also indicated (45, 46). Notably, EDC with intact LMN and FDP with damaged LMN would contraindicate taping or splinting to reach a tenodesis. This statement, however, needs to be proven. Nevertheless, Thomas and colleagues investigated the motoneuron excitability after cSCI and showed that intrinsic motoneuron excitability could change the generation and strength of involuntary muscle contractions (47). External stimulus applied to the muscle spindles, for example, pressure or stretch on the muscle belly, may reinforce this effect (48). Muscle spindles are sensitive length-tension receptors in the skeletal muscles. A stretch-induced activation causes excitation of the Ia and II afferents in the spindle. Hypothetically, splinting or taping the fingers into a flexed position would excite finger extensor afferents. If the EDC has a UMN lesion, the intention to shorten flexors muscles might be hampered by increased activation of the EDC.

Normally, stimulation with long impulses is recommended for the finger flexors. The primary goal is to prevent the development of a claw hand by maintaining the contractility and elasticity of the musculature. Increased reflex activity and/or volitional activity on the EDC with concomitant denervation atrophy on the FDP result in an extension in the metacarpophalangeal joints (MCPs) and concomitant flexion of proximal and distal interphalangeal joints. Improving hand function by means of nerve transfer possibly combined with reconstruction of lumbrical function should be evaluated at an appropriate time.

In addition, special attention needs to be paid to the group of partially innervated muscles in which a UMN and LMN lesion can be detected in the same muscle. With neurophysiological diagnostics and motor point mapping, the mixed lesion of lower and upper motor neuron damage in a single muscle can be determined, but the actual proportions of denervated and innervated regions within the muscle are indefinable. It is important to minimize the risk of selecting partially denervated muscles as donors for muscle–tendon transfers or as recipient muscles in the nerve transfers. In the first case, there may be reduced strength improvement. In the second case, there is a diminished likelihood of reinnervation. Consequently, these muscles should be stimulated both *via* nerves with short and directly *via* muscles with long impulses. This ensures that all parts of the muscle can benefit from the ES according to their damage.

The relatively high number of AIS D with NLI C4 and NLI C5 with intact LMN requires reconsideration of the entire standardized hand positioning scheme. Based on the present results, the patients with AIS C and D with intact LMN develop muscle strength of >3 at 24 weeks after injury. This indicates that splinting and positioning could be removed in favor of immediate task-specific training supported by FES. Not all the muscles show the expected damage to the LMN at the level of segmental innervation. The brachioradialis muscle seems to be an exception. Its segmental innervation is at the level of C5/6. In our survey, only half of the 68.5% of innervated BRs allocate to the area of segmental innervation. Nevertheless, the muscle offers challenges in selective strengthening despite good stimulability and functionality in performing a range of motion exercises.

Regardless of whether traditional therapy or a surgical reconstructive procedure is chosen, electrostimulation *via* muscle with long pulses or stimulation *via* nerve with short pulses for motor learning is recommended. Muscle stimulation aims at maintaining and reversing muscle properties, and nerve stimulation at motor learning and, if necessary, targeted strengthening.

Nerve transfers should be considered early in the post-injury period, especially, if the recipient's muscle has an LMN lesion. Concerns about performing this intervention too early and thus pre-empting and adversely affecting possible neurological recovery are unfounded.

### Limitation

In the present data analysis, two extrinsic hand muscles and one double-jointed forearm muscle were examined. Our selection of muscles studied only reflects one aspect of the complexity in the treatment of the tetraplegic hand. The function and influence of the intrinsic musculature on the function and shape of the hand are not yet included. Nevertheless, the type of motoneuronal damage is most likely a decisive factor and must be included in the treatment and decision-making process, whether surgical or conservative.

## Conclusions

Motor point and AIS in the acute phase after cSCI are important predictors for the further development of muscle tone and strength in the finger flexors and extensors. Therefore, these predictors can enable more accurate guidelines for whom/when not to institute “tenodesis splinting.” Furthermore, the predictors are helpful to create individualized treatment plans that define the appropriate ES from the perspectives of motor learning, strengthening, and preservation of muscle properties.

## Data Availability Statement

The original contributions presented in the study are included in the article/supplementary material, further inquiries can be directed to the corresponding author.

## Ethics Statement

Ethical review and approval was not required for the study on human participants in accordance with the local legislation and institutional requirements. Written informed consent for participation was not required for this study in accordance with the national legislation and the institutional requirements.

## Author Contributions

IB conceived the idea and designed the study. JK performed the statistical data analysis. JF and IB interpreted the data and wrote and revised the manuscript. All authors have verified the underlying data. The final version of the manuscript was read and approved by all authors.

## Funding

Funding for this project was provided by the Swiss Paraplegic Centre, Nottwil, Switzerland.

## Conflict of Interest

The authors declare that the research was conducted in the absence of any commercial or financial relationships that could be construed as a potential conflict of interest.

## Publisher's Note

All claims expressed in this article are solely those of the authors and do not necessarily represent those of their affiliated organizations, or those of the publisher, the editors and the reviewers. Any product that may be evaluated in this article, or claim that may be made by its manufacturer, is not guaranteed or endorsed by the publisher.
